# Effect of Adolescent Obesity on Cardiometabolic Risk in African-Americans and Caucasians

**DOI:** 10.5402/2012/603205

**Published:** 2012-12-04

**Authors:** Robert P. Hoffman

**Affiliations:** Division of Pediatric Endocrinology, Metabolism, and Diabetes, Nationwide Children's Hospital, 700 Children's Drive, Columbus, OH 43205, USA

## Abstract

African-Americans have more hypertension, stroke, and type 2 diabetes than do Caucasians. Endothelial dysfunction and insulin resistance are precursors for each. Since these diseases have origins in pediatrics and are associated with obesity, this study was designed to determine if obesity has different effects on endothelial function, insulin sensitivity, and secretion in African-American and Caucasian adolescents. Thirty-three Caucasian and 25 African-Americans (10–18 years old) were subdivided by BMI into lean, overweight, and obesity groups. Endothelial function was measured as forearm vascular resistance (FVR) over 1 min following 5 min of upper arm vascular occlusion. Insulin sensitivity and secretion were measured using intravenous glucose tolerance test and minimal model. Postocclusive FVR was significantly increased in obese African-Americans. Insulin sensitivity was reduced in obese subjects but did not differ by race. Insulin secretion was increased in African-Americans but did not differ by obesity. Subjects were subdivided into risk groups based on 20th percentile for postocclusion FVR response in lean. Seven of nine obese African-Americans were in the high risk group compared to 0 of 5 obese Caucasians. These results demonstrate that obesity significantly impairs endothelial function in African-Americans. Endothelial dysfunction likely predisposes to future cardiometabolic disease in obese African-American adolescents.

## 1. Introduction

African-Americans have increased incidence of and mortality from cardiometabolic diseases, including stroke [1–3], hypertension [4], and type 2 diabetes [4–6]. The incidence of myocardial infarction among African-Americans may be lower than among Caucasians [7], but mortality is increased in African-Americans, particularly at younger ages. Hypertension, stroke, type 2 diabetes, and myocardial infarction are frequent outcomes of obesity and the metabolic syndrome. Their increased frequency in African-Americans is surprising since the metabolic syndrome is less common in African-Americans than in Caucasians [4]. This raises the question of whether there are racial differences in obesity's effects on nontraditional cardiometabolic risk factors that are not part of the metabolic syndrome. 

One such factor is endothelial function. Endothelial function is closely related to insulin sensitivity, and African-Americans are frequently insulin resistant compared to Caucasians, both in adulthood and adolescence [8–10]. Insulin causes endothelially mediated vasodilation [11], and increases in nutritive blood flow in response to insulin are associated with increased limb glucose uptake in animals [12]. Diminished endothelial function decreases insulin-induced vasodilation and glucose delivery to and usage by muscle tissue and predisposes to insulin resistance, and type 2 diabetes, and other cardiometabolic diseases. There is considerable evidence that African-Americans have altered endothelial function. Levels of endothelin 1, an endothelially secreted vasoconstrictor, are increased in African-Americans [13], and flow mediated brachial artery vasodilation is diminished, as well [14]. Hinderliter et al. [15, 16] found higher minimum forearm vascular resistance (FVR) following vascular occlusion in African-American, young adults than in similar aged, Caucasian subjects.

Atherosclerotic disease and type 2 diabetes have their origins in childhood and adolescence [17, 18]. It is, therefore, of critical importance, if we are to understand increased cardiometabolic risk in African-Americans that we study this age group. Prepubertal, pubertal, and postpubertal African-American adolescents have increased insulin secretion and impaired endothelial function compared to Caucasian adolescents [19]. The goal of this study is to examine racial differences in endothelial function responses to obesity in African-American and Caucasian adolescents by reexamining previously reported data [19] subdivided into three obesity categories: lean, BMI < 85%; overweight, 85% ≤ BMI < 95%; obese, BMI ≥ 95%.

## 2. Methods

### 2.1. Subjects

Subject characteristics have been previously reported [19]. Briefly, 58 (25 African-American and 33 Caucasian) healthy, adolescent volunteers between 8 and 18 years of age were recruited for participation by advertisement. There were no weight criteria. They were taking no medications and were free from chronic and acute disease at the time of study. Pregnancy tests were done on all menstruating females and were negative. The study was approved by the Ohio State University Office of Responsible Research. Informed consent was obtained from a parent or legal guardian and assent from each subject. 

### 2.2. Protocol

 Subjects were admitted to the Clinical Research Center of the Ohio State University after an overnight fast. Upon arrival endothelial function was assessed using vascular occlusion plethysmography to measure forearm blood flow (FBF) for two minutes before and one minute after five minutes of upper arm arterial occlusion (200 mmHg) [19]. FVR was calculated by dividing mean arterial pressure by FBF. Endothelial function was assessed as the postocclusive FVR and percent change in FVR from pre- to postocclusion.

After completion of the test of endothelial function, intravenous catheters were placed in each arm for the frequently sampled, intravenous glucose tolerance test to obtain the fasting blood sample for measurement of the fasting lipid profile. The total glucose dose was 250 mg/kg. Three milliliter blood samples were taken at −10, 0, 2, 4, 6, 8, 12, 14, 16, 19, 22, 27, 32, 42, 52, 62, 72, 82, 92, 102, 122, 142, 162, and 182 minutes relative to a glucose bolus for measurement of plasma glucose and insulin concentrations. Total body insulin sensitivity (*S*_*I*_) was calculated using the one compartment minimal model (Minmod, Minmod Inc., Los Angeles, CA, USA). The program was also used to calculate the acute insulin response to glucose (AIRG) over the first 19 minutes of the test.

### 2.3. Assays

Plasma glucose and insulin concentrations were measured in the CORE lab of the Clinical Research Center of the Ohio State University. Plasma lipids were measured in the Clinical Laboratory of the Ohio State University Hospital.

### 2.4. Statistical Analysis

Subjects were subdivided by race and BMI category (lean, BMI < 85%; overweight, 85% ≤ BMI < 95%; obese, BMI ≥ 95%). Analysis of variance was used to assess racial and obesity group differences. Planned contrasts were used for posthoc analysis. Secondarily, data from lean subjects was used to determine the 20th percentile for endothelial function and insulin sensitivity. Subjects with values above the 20% for either endothelial function or insulin sensitivity were considered at low risk, while subjects with values below the cutoff were considered at high risk. Chi-squared analysis was used to determine racial differences in frequencies for obese subjects in the high and low risk groups and *t* tests were used to compare differences in blood pressure and lipids between high and low risk groups. The Bonferroni method was used for correction of multiple comparisons. Data were log normalized, as needed. Differences were considered significant at *P* < 0.05, and tendencies are mentioned as *P* < 0.1. 

## 3. Results

### 3.1. Subject Characteristics

Table 1 shows the number of subjects and mean age, BMI, blood pressure, and lipids for each of the race and obesity groups. Age did not differ between the groups by race or obesity. BMI was not different between the races. Systolic blood pressure was significantly increased in African-Americans (*P* = 0.02) and was not affected by obesity. There was no race by obesity interaction. Obesity grouping significantly affected diastolic pressure (*P* = 0.03, Table 1) with diastolic pressure in the combined overweight groups being significantly greater than in the combined obese groups (*P* = 0.027). For lipid measurements, only the obesity effects were significant or near significant (log triglyceride, *P* = 0.004; LDL, *P* = 0.097; HDL, *P* = 0.008). Triglycerides levels in the combined obese groups were significantly greater than in lean (*P* = 0.003) or overweight (*P* = 0.045). For LDL none of the between group specific comparisons were significant. HDL tended to be lower in combined obese compared to lean subjects (*P* = 0.075) and was significantly lower in obese than in overweight (*P* = 0.009).

### 3.2. Endothelial Function and Insulin Sensitivity and Secretion

**Figure 1:**
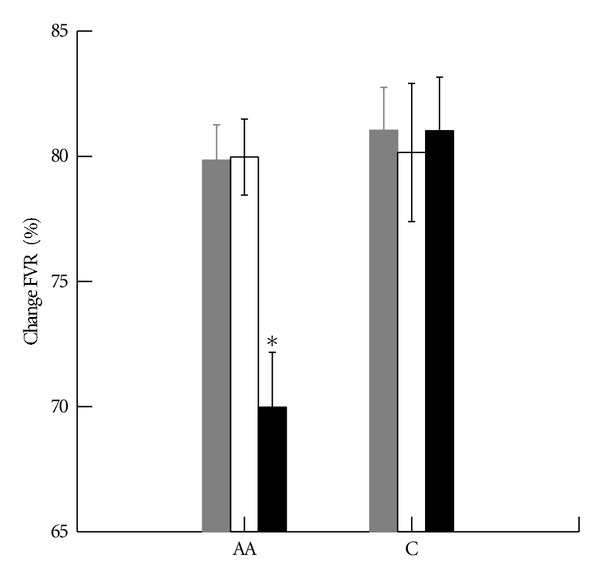
Percent change in forearm vascular resistance following upper arm occlusion in adolescents. Lean gray; overweight open; obese solid. *P = 0.023 versus African-American lean and 0.068 versus Caucasian obese. Multiple comparisons used Bonferroni correction. Error bars represent SE.

For postocclusion FVR (Table 2), analysis of variance with preocclusion FVR as a covariate revealed significant race (*P* = 0.021), obesity effects (*P* = 0.020), and, most importantly, a significant race by obesity interaction (*P* = 0.034). Subgroup comparisons revealed that postocclusion FVR in obese African-Americans was significantly greater than in African-American lean (*P* < 0.001) or overweight (*P* = 0.015) and, also, greater than in Caucasian obese subjects (*P* = 0.003). No racial differences were present between African-American and Caucasian lean and overweight subjects, and no weight group differences were present for Caucasian subjects. For percent change in FVR (Figure 1), analysis of variance revealed near significant race (*P* = 0.05), obesity effects (*P* = 0.089), and race by obesity interaction (*P* = 0.069). Between group comparisons, again, revealed significantly diminished endothelial function only in the obese African-American group (*P* = 0.023 versus African-American lean and *P* = 0.068 versus Caucasian obese).

**Figure 2:**
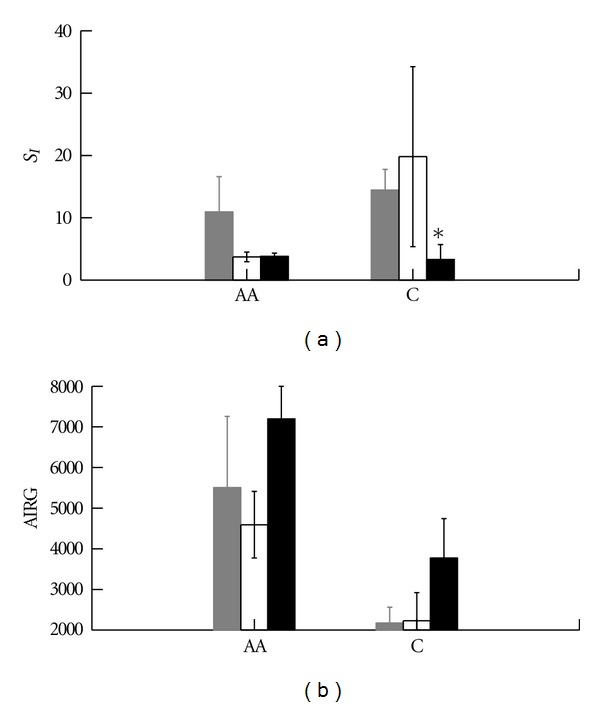
Frequently sampled intravenous glucose tolerance test results in African-American and Caucasian adolescents. Insulin sensitivity index (*S*_*I*_, l mU^−1^ min^−1^); obese less than lean African-American and Caucasian combined *P* = 0.048, obesity race interaction *P* = 0.018. *P < 0.001 versus Caucasian lean, *P* = 0.012 versus Caucasian overweight, and *P* = 0.084 versus African-American obese. Multiple comparisons used Bonferroni correction. Error bars represent SE. Acute insulin response to glucose (AIRG, uU min ml^−1^). Lean gray; overweight open; obese solid. African-American significantly greater than Caucasian in all subjects, *P* = 0.007. No significant specific between group differences. Error bars represent SE.

For log *S*_*I*_ (Figure 2), the overall race effect was not significant, but the obesity effect (*P* = 0.018) and race by obesity interactions (*P* = 0.021) were significant. Log *S*_*I*_ was significantly lower in Caucasian obese than in Caucasian lean (*P* < 0.001) and overweight (*P* = 0.012) and tended to be less than in AA obese (*P* = 0.084). For log AIRG only the racial effect was significant (*P* = 0.007) with higher insulin secretion in African-American subjects.

### 3.3. High versus Low Risk

For endothelial function 7 of the 14 obese subjects had percent change FVR values below the 20% for lean and were considered high risk. All seven were African-American meaning 7 of 9 obese, and African-American subjects were high risk for endothelial function compared to 0 of 5 obese, Caucasian subjects (*P* = 0.005). Obese subjects in the high risk group had a tendency toward increased insulin secretion compared to low risk subjects (*P* = 0.054), but there were no differences in age, BMI, blood pressure, lipids, or insulin sensitivity between high risk and low risk subjects. For nonobese subjects there were no racial differences in frequency of high versus low risk subjects. No differences were found between low and high risk, nonobese subjects.

For *S*_*I*_ 8 of 13 obese subjects were in the high risk group (*S*_*I*_ data not available in 1 obese subject). For obese subjects there was no racial difference in risk frequency (African-American 5 of 9 high risk, Caucasian 3 of 4 high risk), nor there was a racial frequency difference between high and low risk for nonobese subjects. In obese subjects there were no differences for age, BMI, lipids, blood pressure, and endothelial function between high and low risk subjects based on *S*_*I*_. For nonobese subjects BMI was greater in high risk than low risk subjects. 

Four of the nine obese African-American subjects were high risk for both endothelial function and *S*_*I*_. No differences were found in age, BMI, blood pressure, and obese subjects in the combined high risk group versus those not in the combined group. Five of 38 nonobese subjects were in the combined high risk group, and there was no difference in racial frequency of combined high risk. There was a trend toward increased LDL cholesterol in the combined nonobese high risk group (106 ± 3 versus 92 ± 4 mg/dL; *P* = 0.079). 

## 4. Discussion

These results demonstrate that obesity has a greater impact on endothelial function in African-American adolescents than in Caucasian adolescents. Overall, postocclusive FVR was increased and percent change in FVR was significantly decreased only in the obese African-American group compared nonobese African-American and obese Caucasian adolescents. This is further emphasized by the fact that 7 of 9 obese African American adolescents had endothelial function below the 20% for lean subjects while none of the 5 obese Caucasians were in the high risk group. 

The racial differences in endothelial function could not be accounted for by any of the other measured variables. Demographically, age and BMI were not different between the African-American and Caucasian obese subjects, and there were no differences in plasma triglyceride, LDL, or HDL cholesterol levels between obese African-American and Caucasian subjects. As in the previous report of this data [19], insulin secretion in response to intravenous glucose was significantly greater in African-American than in Caucasians subjects. It was hypothesized, in that report, that increased insulin secretion may account for the impaired endothelial function in the African-American subjects since endothelial function decreased as insulin secretion increased in African-American, but not Caucasian, subjects. This does not appear to be the case in the current study because although AIRG was significantly higher in lean and overweight African-American compared to Caucasian adolescents, endothelial function did not differ between the two races in these weight categories. It is possible that there may be some sort of threshold above which increased insulin secretion manifests its effects since AIRG was highest in obese African-Americans, although the increase was not significantly different from the other African-American groups. 

In this age group, the impaired endothelial function in obese African-American subjects did not yet have any specific clinical outcomes. Systolic blood pressure was significantly higher in African-American adolescents, but this effect was not limited to obese subjects. *S*_*I*_ was lower in obese subjects, but this effect was not different between African-American and Caucasian subjects. Surprisingly, *S*_*I*_ actually tended to be lower in obese Caucasians than in obese African-American subjects. Past studies of adolescent racial differences in insulin sensitivity have found variable results with many finding decreased insulin sensitivity in African-Americans [20]. 

Not all obese subjects have increased risk for cardiometabolic disease. Data from the NHANES study demonstrates that slightly over 30% of obese adults are metabolically healthy [21] and thus have minimal cardiovascular and diabetes risk. Because of this, in the current study obese subjects were divided into high and low risk subjects based on endothelial function and/or insulin sensitivity below the 20% for lean subjects. This level was chosen since not all lean subjects are at low cardiometabolic risk. Comparisons were then made between high risk and low risk subjects. Interestingly, 7 of the 9 obese African-Americans were in the high risk group while none of the 5 Caucasians were. This finding clearly indicates that obesity increases endothelial risk in African-Americans compared to Caucasians. When comparisons were made between high and low risk subjects for endothelial function the only difference that approached significance was increased AIRG. This may simply be related to the fact that the high risk group was exclusively African-American. No racial differences or specific risk factors were found for risk group differences in obese subjects based on insulin sensitivity. For lean subjects increased BMI was associated with increased insulin sensitivity risk. This is not surprising but does indicate that even small increases in BMI within the normal range are associated with increased insulin resistance. LDL cholesterol tended to be increased in lean subjects in combined high risk group for both endothelial function and insulin sensitivity. This is the only place any of the lipid measurements was associated with increased cardiometabolic risk in this study. This speaks to the need for future studies of adolescent cardiometabolic risk to go beyond traditional risk factors. 

The major limitation of this study is the small sample size, particularly in the obese group. The small sample size may limit our ability to detect significant differences between racial and obesity groups. However, a significant decrease in endothelial function was still detected in obese African-American adolescents compared to obese Caucasian and lean and overweight African-Americans. The small size is more likely to impair our ability to find differences in lipids and blood pressure between high and low risk groups particularly when applying the Bonferroni method of correcting for multiple comparisons. If the Bonferroni method is not applied, HDL cholesterol is surprisingly significantly higher in obese subjects at high endothelial risk compared to those at low endothelial risk. For nonobese subjects, triglyceride levels are increased in high endothelial risk subjects. For insulin sensitivity, both systolic and diastolic blood pressure are increased in non-obese, high risk subjects.

 In conclusion, this study demonstrates that obesity has a greater impact on endothelial function in African-American compared to Caucasian adolescents, which is likely to predispose obese African-American adolescents to higher rates of future cardiometabolic disease. Future studies of cardiometabolic risk in adolescent obesity will need to account for these racial differences and explore potential mechanisms and preventions for this effect. 

## Figures and Tables

**Table 1:** Subject characteristics according to race and obesity group (mean ± SE).

	Lean		Overweight		Obese	
	African- American	Caucasian	African- American	Caucasian	African- American	Caucasian
Number	12	22	4	6	9	5
Age (years)	13.4 ± 1.0	13.2 ± 0.7	12.5 ± 1.5	13.5 ± 0.8	12.8 ± 0.9	15.4 ± 1.3
BMI kg m^−2^	21.0 ± 0.8	19.7 ± 0.7	22.3 ± 0.8	24.6 ± 1.0	28.8 ± 1.4	28.0 ± 0.8
Systolic blood pressure (mmHg)	112 ± 4	104 ± 2	122 ± 1	109 ± 3	110 ± 5	105 ± 6
Diastolic blood pressure (mmHg)	58 ± 3	53 ± 1	62 ± 3	59 ± 2	51 ± 3	51 ± 3
Triglyceride (mg/dL)	54 ± 10	48 ± 4	44 ± 6	55 ± 6	72 ± 14	112 ± 30
LDL (mg/dL)	97 ± 8	89 ± 4	99 ± 11	108 ± 9	108 ± 7	106 ± 7
HDL (mg/dL)	47 ± 4	50 ± 3	62 ± 9	52 ± 5	42 ± 5	34 ± 2

**Table 2:** Postocclusion FVR (mmHg dL min/mL) in adolescents according to race and obesity status (mean ± SE).

	Lean	Overweight	Obese
African-American	5.2 ± 0.4	5.1 ± 0.7	8.5 ± 1.6*
Caucasian	3.9 ± 1.6	5.1 ± 0.6	4.0 ± 1.0

*P = 0.003 versus Caucasian obese; *P* < 0.001 versus African-American lean; *P* = 0.015 versus African-American overweight.
